# Deep ultraviolet ultrashort laser pulses for precise ablation of soft biological tissue

**DOI:** 10.1364/BOE.578629

**Published:** 2025-12-10

**Authors:** Tatiana K. Malikova, Rainer J. Beck, Timothy P. Frazer, Paul M. Brennan, Kevin Dhaliwal, Robert R. Thomson, Jonathan D. Shephard

**Affiliations:** 1 Institute of Photonics and Quantum Sciences, Heriot-Watt University, Edinburgh, EH14 4AS, UK; 2 Translational Neurosurgery, Centre for Clinical Brain Sciences, University of Edinburgh, Edinburgh, EH16 4SB, UK; 3 Centre for Inflammation Research, Institute for Regeneration and Repair, University of Edinburgh, Edinburgh, EH16 4UU, UK

## Abstract

Laser ablation offers the potential for precisely removing pathological tissue without damaging surrounding healthy structures. Among the existing laser types, deep ultraviolet ultrashort pulsed lasers offer the highest axial precision and reduced collateral damage, yet their application for ablating soft tissues apart from the cornea remains underexplored. Here, ablation of *ex vivo* lamb liver using laser pulses at 206 nm wavelength and 250 fs pulse duration is investigated. Laser parameters that enable clean, controlled tissue removal are identified by systematically varying the laser pulse energy, spot size, and pulse repetition rate, and the ablated tissues are analysed using histological analysis and surface profilometry. With optimised settings, tissue removal with axial precision down to 10 microns is demonstrated. Ablation threshold fluence of 38.7 ± 2.1 mJ·cm^−2^ is determined for lamb liver tissue, and fluence windows yielding precise ablation with no observable collateral damage are defined for different laser spot sizes. The ablation responses of tissues with different physical properties are also investigated. These results advance understanding of laser-tissue interaction in the deep ultraviolet ultrashort pulse regime and demonstrate the potential of the proposed tissue removal method for high-precision surgical applications.

## 1. Introduction

Precise removal of pathological tissue while preserving healthy structures is one of the key objectives in modern surgery. This is particularly important in tumour resection, where maximal removal of malignant tissue has been linked to lower recurrence rates and improved patient survival [1,2]. However, in anatomically complex or sensitive organs such as the brain, complete resection is not always possible, as tumours can grow around critical structures like blood vessels or nerves [3]. In such cases, technologies that enable highly localised, minimally invasive removal of soft tissue are highly desired. Laser-based surgical techniques, for example laser ablation, are a promising approach, with the potential for fine spatial control and minimal collateral damage [4]. In this work, *laser ablation* is defined as physical removal of target material, as opposed to purely thermal approaches that induce necrosis without removing tissue.

Laser radiation enables several distinct mechanisms for precise tissue removal, each with specific advantages and limitations [5,6]. Most surgical lasers, such as the ${\mathrm{CO}}_{2}$ laser, rely on photothermal ablation, where laser energy is absorbed by tissue and converted into heat. This regime allows for simultaneous cutting and coagulation, which can be used for haemostasis during surgery in blood-rich organs [7,8] but can be detrimental when operating around critical structures. Another mechanism is plasma-mediated ablation, where ultrashort (<10 ps) laser pulses – typically in near-infrared (NIR) wavelength range – generate plasma by ionising target material. The rapid plasma expansion generates a shockwave that can drive cavitation or explosive tissue removal [6]. Since this approach relies on nonlinear absorption, the use of ultrafast NIR pulse for tissue ablation is particularly well suited for precise surgery in optically transparent tissues. A notable example is SMILE vision correction surgery, where NIR femtosecond pulses create highly localised incisions within the cornea [5]. Plasma-mediated ablation has also been explored for applications in vocal fold surgery [9,10], resection of bowel tumours [11], and bone spur removal [12]. A third distinct regime – photochemical decomposition – is driven by deep ultraviolet (deep-UV, λ < 280 nm) photons which, due to their high energy, can directly break molecular bonds in the tissue. Strong absorption of deep-UV light by proteins, water, and DNA confines the interaction zone to a few micrometres, enabling excellent axial ablation precision [6]. This regime is employed in LASIK surgery, where a nanosecond 193-nm ArF excimer laser removes corneal tissue with sub-micrometre precision [13].

Compared to photothermal and plasma-mediated regimes, photochemical ablation appears most suitable for minimally invasive, micrometre-scale tissue removal. First, the recoil stress generated by deep-UV ablation products is relatively modest (8-15 MPa [14]) compared to GPa-scale shockwaves induced by plasma expansion during NIR ultrashort pulsed laser ablation [6]. Therefore, ablation by deep-UV light potentially causes less disruption to the surrounding tissue. Additionally, plasma-mediated ablation requires high intensities typically achieved by tight focusing, resulting in short Rayleigh ranges (tens to hundreds of microns). This in turn means that NIR ultrashort pulsed ablation process is highly sensitive to defocusing: slight axial misalignments can cause subsurface cavitation or ablation failure [15,16]. In contrast, deep-UV ablation occurs only at the tissue surface and is therefore far less sensitive to beam focus variations. Finally, the shallow penetration of deep-UV radiation into the tissue limits heat diffusion and minimises collateral thermal damage, as opposed to photothermal ablation regime.

To further enhance ablation precision, deep-UV radiation can be delivered in ultrashort pulses, confining heat deposition and stress propagation more tightly to the irradiation zone. In theory, this could offer the highest ablation precision among all laser-tissue interaction regimes. Despite this, studies investigating ultrashort pulsed deep-UV lasers for soft tissue removal remain limited. Vengris *et al.* and Danieliene *et al.* examined femtosecond deep-UV laser ablation of cornea both *ex vivo* and *in vivo*, discovering that the outcomes of refractive surgery performed with the fifth harmonic (206 nm) of a 1030-nm solid-state femtosecond laser were comparable to those of a surgical 193-nm ArF excimer nanosecond laser in terms of precision, operative time, and healing [17–19]. Bille *et al.* demonstrated precise *ex vivo* brain resection using 211-nm picosecond laser pulses, with the aim of maximising ablation depth and efficiency while minimising collateral damage [20]. However, they obtained similar results with NIR ultrashort pulses [21] and ultimately implemented the NIR system in a neurosurgical probe prototype [15].

To fully benefit from the potential of deep-UV ultrashort pulsed lasers for precise soft tissue removal, and to enable future surgical applications, comprehensive studies are essential. In particular, characterising ablation outcomes across a range of laser parameters and tissue types is critical, as thermomechanical properties can strongly influence ablation kinetics [6]. This study investigates ablation of a soft biological tissue model using deep-UV femtosecond laser pulses and examines the role of laser pulse energy, spot size, and pulse repetition rate in the ablation process, aiming to identify regimes that maximise precision and minimise collateral damage. As a practical first step toward translation to more clinically relevant and less accessible biological tissues such as brain, the experiments are performed in lamb liver – soft, mechanically weak water-rich biological tissue with low collagen content [6].

## 2. Materials and methods

### 2.1. Laser source, beam delivery, and tissue irradiation

**Fig. 1.**
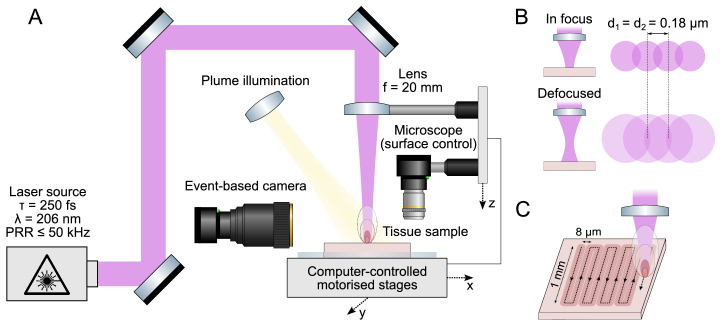
**Experimental setup and methodology for laser ablation. A**: Schematic of the setup for irradiation of biological tissue using deep-UV laser pulses and visualisation of the ablation plume. **B**: Changing the effective laser spot size on the tissue surface by moving the focal plane above the sample surface. Strong absorption at 206 nm enables efficient ablation even out of focus. **C**: Schematic of the raster scan pattern used throughout the experiments.

The experimental setup for biological tissue ablation is shown in Fig. 1(A). The laser source (Pharos, Light Conversion) produces pulses of 250 fs duration at a fundamental wavelength of 1030 nm. These are converted to the fifth harmonic (206 nm) using an integrated nonlinear frequency conversion module. The maximum pulse repetition rate (PRR) of the laser source at 206 nm is 50 kHz and can be reduced via an internal pulse picker.

The approximately Gaussian laser beam (0.52 ± 0.08 mm spot diameter at $1\cdot e^{-2}$ intensity level) is directed towards the ablation site using UV-enhanced silver coated mirrors and focused onto the sample surface using a plano-convex calcium fluoride lens. The focusing lens is mounted on a computer-controlled vertical motorised stage (Aerotech) which adjusts the axial position of the focal point relative to the sample surface. The beam profile and spot size at focus were characterised using a knife-edge technique, with the spot size defined at the $1\cdot e^{-2}$ intensity level. The measured spot diameter in focus was 12.8 ± 1.0 μ m (20-mm focal length lens, NA 0.006); spot diameter will be referred to as “spot size” throughout the manuscript. The maximum laser pulse energy after the focusing optics was 1.09 ± 0.06 μ J. The pulse energy is controlled using an integrated variable attenuator. Lateral positioning of the beam on the sample surface is achieved by translating the sample in the XY plane using motorised stages (Aerotech). A microscope with a camera is also mounted onto the Z-axis stage to facilitate precise positioning of the tissue surface relative to the laser focus.

### 2.2. Methodology of ablation experiments

Lamb liver was selected as an accessible soft tissue model that offers high reproducibility across samples, which is essential for isolating the effects of laser scanning parameters. Liver is a water-rich, collagen-poor tissue [6], making it mechanically weak compared to other readily available animal tissues commonly used in laser ablation studies, such as chicken breast [22–24]. Lamb liver tissue samples (sourced from a local supermarket) were cut into rectangular sections approximately 2 × 1 ${\mathrm{cm}}^{2}$ in area and 0.5 cm thick. Individual sections were then transferred to the irradiation setup for laser processing. To minimise drying effects, the time between the beginning of tissue preparation and the end of ablation experiments was kept under 15 minutes.

The following parameters could be varied during the ablation experiments: laser pulse energy ( E ), laser PRR, which determines temporal pulse separation, spatial separation of successive laser pulse impact points on the tissue surface (laser spot spatial separation, d ), and effective laser spot size on the tissue surface ( D ). Pulse energy and PRR were directly controlled via the laser software interface. Spatial separation between laser spots was determined by the speed of the translation stages during laser beam scanning, taking into account the laser PRR. To adjust the effective laser spot size on the tissue surface, the focal plane was positioned above the sample surface – an approach justified by the surface-mediated nature of deep-UV ablation process (Fig. 1(B)). Peak fluence, characterising laser pulse energy of a Gaussian beam delivered per unit area, was defined as:

(1) $$F_{p}=\frac{2E}{\pi {\left(\frac{D}{2}\right)}^{2}}$$

Because the laser pulse duration (250 fs) is orders of magnitude shorter than the interval between pulses, even at the highest PRR used (50 kHz), single-pulse peak fluence was chosen as the relevant metric for characterising ablation outcomes, rather than the accumulated energy per unit area resulting from multiple overlapping pulses in the raster scan.

At 206 nm, the characteristic optical penetration depth in biological tissue is on the order of 1 μ m [6,25], which is smaller than the natural surface profile variations of the samples ( ∼ 10 μ m). The effective spot size change due to these variations is also well below the uncertainty of the spot size measurements. Therefore, variations in focusing depth or beam propagation have a negligible influence on the fluence delivered to the surface.

A raster scan pattern was selected for ablation outcome analysis (Fig. 1(C)). The pattern consisted of 30 lines, each 1 mm in length, spaced 8 μ m apart. This spacing corresponds to the ∼ 2/3 of the minimal spot size (12.8 μ m), ensuring full coverage of the sample surface during ablation. The distance between successive laser pulse impact points was fixed at 0.18 μ m for all experiments, independent of the laser spot size, as illustrated in Fig. 1(B). At 1 kHz PRR, this corresponded to 0.18 mm/s sample scan speed. This high degree of laser spot overlap was intentionally applied to amplify potential interactions between successive ablation events and to identify laser parameters that minimise collateral damage under these restrictive conditions. The total number of pulses delivered per unit area was very high ( ∼ 170,000 pulses per 1 mm × 0.24 mm rectangle), but remained the same across all reported experimental configurations reported in this study, independent of spot size or PRR, which allowed direct comparison of single-pulse effects between different parameter sets.

To determine the ablation threshold, a high-speed event-based (neuromorphic) camera (EVK4HD, Prophesee) was used. This type of camera is highly sensitive to local changes in brightness, making it well-suited for detecting transient phenomena such as the onset and dynamics of ablation plumes – the clouds of particles ejected from the sample during ablation. The camera was positioned parallel to the tissue surface, while a bright light source illuminated the ablation site at a 45-degree angle (Fig. 1(A)). The ablation plume, generated by irradiating a stationary tissue sample for one second using a PRR of 1 kHz, was imaged using the event-based camera. After each measurement, the lateral position of the laser spot on the tissue surface was changed. To determine the threshold fluence, these measurements were performed across a range of laser pulse energies and effective spot sizes. For each fixed spot size, the pulse energy was gradually reduced until the plume signal was no longer detected; the lowest fluence at which a plume was still observed was recorded. These spot-size-specific fluence values were then averaged to obtain the final reported ablation threshold fluence. This method specifically reports the peak fluence, because ablation occurs when the peak of the Gaussian beam profile exceeds the threshold. A similar approach for determining ablation thresholds, based on the detection of light scattered by ablation plume particles, was previously used by Bille *et al.* [20] and Loesel *et al.* [26].

### 2.3. Post-ablation tissue analysis

Immediately after laser irradiation, lamb liver samples were analysed using an optical surface profilometer (InfiniteFocus, Alicona), which provided surface topography measurements with an axial resolution of 0.3 μ m and transverse resolution of 2.9 μ m. These data were used to quantitatively determine the depth of the ablation cavities. Optical images of the ablated areas were also obtained. The samples were then fixed in 10% neutral-buffered formalin for 48 hours to enable histological processing.

Histological analysis was performed to evaluate the effects of laser ablation on tissue morphology and to identify any areas of collateral damage. Fixed samples were dehydrated, embedded in paraffin, sectioned into 4-micron slices, and stained with haematoxylin and eosin (H&E). As these processing steps can introduce sample deformation, the resulting sections were used only for qualitative assessment of laser-induced collateral damage. All histology procedures were carried out by SURF Histology Facility at the University of Edinburgh.

One-way ANOVA (significance level p = 0.05), followed by post-hoc Tukey HSD tests was performed on ablation cavity depth measurements to assess reproducibility across different tissue samples. Each data point represented the mean cavity depth from one tissue sample. All statistical analyses were carried out in Python (statsmodels library).

## 3. Results

### 3.1. Ablation threshold measurement

The ablation threshold in lamb liver tissue was measured using a neuromorphic camera, as described in Section 2.2. Across a range of spot sizes (12.8 μ m to 64.6 μ m), the lowest fluence that produced a detectable ejecta plume remained approximately constant, even though the absolute energy required for ablation varied significantly. For example, at a spot size of 12.8 μ m, the minimum energy required to produce an ablation plume was 0.02 μ J, corresponding to a fluence of 32 $\mathrm{mJ}\cdot {\mathrm{cm}}^{-2}$ . In contrast, for a 64.6 μ m spot size, a much higher energy of 0.52 μ J was needed to reach the same fluence and generate a plume. This trend confirms that the onset of ablation is governed by fluence, and not by absolute energy. The ablation threshold fluence was found to be 38.7 ± 2.1 $\mathrm{mJ}\cdot {\mathrm{cm}}^{-2}$ .

**Fig. 2.**
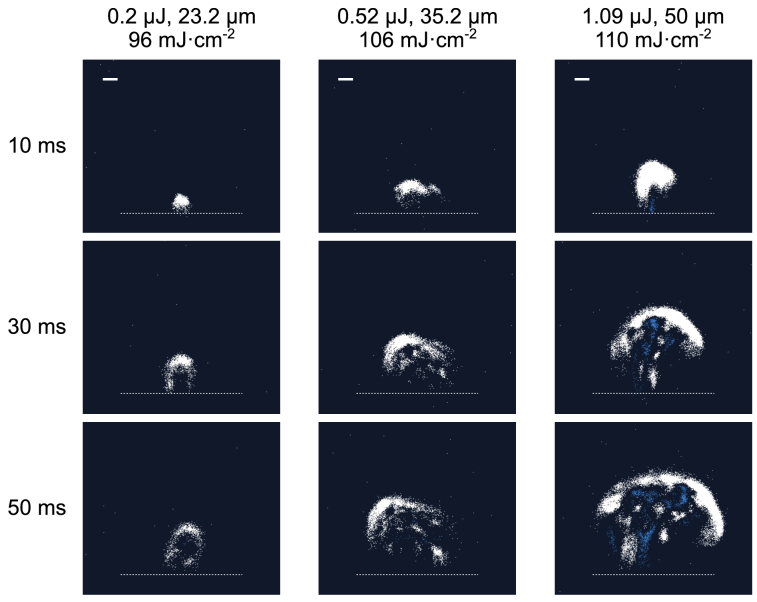
**Images of the ablation plumes produced by different combinations of laser pulse energy and spot size yielding similar peak fluences.** Plume size scales with laser pulse energy. Ablation was performed at 1 kHz pulse repetition rate, with the tissue fixed under the laser beam (ablation of craters). Images were captured by an event-based camera 10, 30 and 50 ms after the first laser pulse income; scale bar (bold white line in top left corner of top-row images) is 200 μ m. The dotted line at the bottom marks the tissue surface. For footage of dynamic plume behaviour across different laser settings, see **Visualization 1, Visualization 2 and Visualization 3**. Left column: laser pulse energy E = 0.2 μ J, spot size D = 23.2 μ m, peak fluence $F_{p}$ = 96 $\mathrm{mJ}\cdot {\mathrm{cm}}^{-2}$ . Middle column: E = 0.52 μ J, D = 35.2 μ m, $F_{p}$ = 106 $\mathrm{mJ}\cdot {\mathrm{cm}}^{-2}$ . Right column: E = 1.09 μ J, D = 50 μ m, $F_{p}$ = 110 $\mathrm{mJ}\cdot {\mathrm{cm}}^{-2}$ .

The use of different combinations of laser energy and spot size yielding the same peak fluence value in the range between 70 and 500 $\mathrm{mJ}\cdot {\mathrm{cm}}^{-2}$ did not produce identical ablation plumes. While the threshold for ablation plume formation depended solely on fluence, plumes were larger and more intense during ablation at higher energies and larger spot sizes (Fig. 2 and Visualization 1, Visualization 2 and Visualization 3). At peak fluences close to the ablation threshold value (40 – 70 $\mathrm{mJ}\cdot {\mathrm{cm}}^{-2}$ ), the difference between the plumes was less pronounced; plumes at the ablation threshold fluence were the least intense, and consisted of a small number of individual particles.

### 3.2. Effects of laser pulse energy and spot size on ablation results

**Fig. 3.**
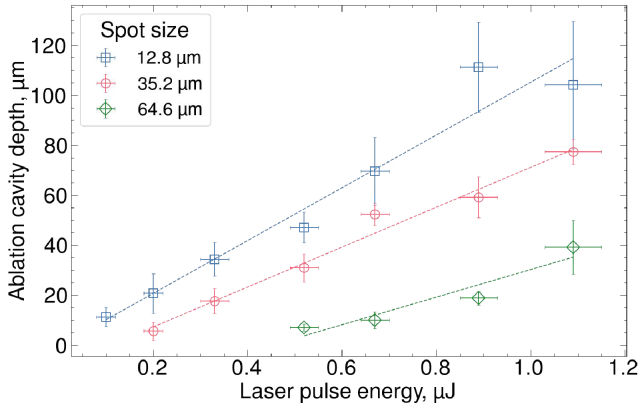
**Ablation cavity depth as a function of laser pulse energy for three different laser spot sizes**: 12.8 μ m (blue squares), 35.2 μ m (red circles), and 64.6 μ m (green diamonds). For each spot size, increasing the laser pulse energy leads to deeper ablation cavities. At the same pulse energy, smaller spot sizes result in greater ablation depth. Measurements were performed in fresh tissue sample immediately after ablation. Each data point represents a single ablation cavity, with vertical error bars showing the standard deviation of the depth measurements within that cavity; uneven surface profiles of cavities produced at high energies contribute to large depth measurement deviations.

To investigate the role of laser pulse energy and laser spot size in the ablation outcome, a systematic parameter field study varying both parameters was conducted. For each combination of pulse energy and spot size, a single ablation cavity was produced in a fresh tissue sample and its depth measured using a surface profilometer immediately after ablation. Multiple depth measurements were acquired at different positions across the cavity floor, and the cavity depth is reported as the mean and standard deviation of these measurements. All cavities in this dataset and in the following quantitative plots were produced sequentially using segments from a single biological sample, unless stated otherwise – this approach was chosen to minimise inter-sample variability and isolate parameter-dependent trends. The results of these measurements are presented in Fig. 3, which shows the relationship between ablation cavity depth and laser pulse energy for three effective spot sizes at the tissue surface (12.8 ± 1 μ m, 35.2 ± 4.8 μ m, and 64.6 ± 9.2 μ m). These correspond to Gaussian beam waists located at the tissue surface, 1 mm above, and 2 mm above, respectively, as schematically illustrated in Fig. 1(B). In this experiment series, laser pulse energy was decreased from a maximum of 1.09 μ J down to 0.2 μ J, or until no ablation was observed. For all three examined effective spot sizes, irradiation with higher laser pulse energy produced deeper ablation cavities. Furthermore, if the laser pulse energy was constant, ablation cavities were deeper when smaller spot sizes were used.

**Fig. 4.**
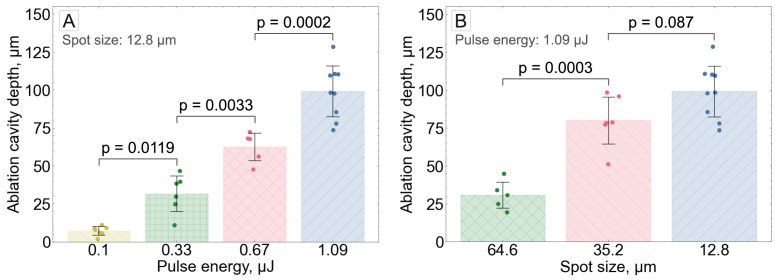
**Statistical analysis of ablation cavity depth across multiple tissue samples. A**: Effect of laser pulse energy on cavity depth at a constant effective spot size of 12.8 μ m. **B**: Effect of effective spot size on cavity depth at a constant pulse energy of 1.09 μ J. Each data point represents the mean ablation cavity depth measured within a single tissue sample. Bars show the mean of these sample means (n = 5–9 samples, depending on irradiation parameters), and error bars indicate the standard deviation across samples. Brackets above the bars indicate pairwise comparisons from the Tukey HSD test, with the corresponding p-values provided above the brackets.

To evaluate whether these trends were consistent across different tissue samples, a broader dataset comprising measurements collected at multiple stages of the project was analysed (Fig. 4). Analysis of variance (n = 5–9 samples, depending on irradiation parameters) showed that both laser pulse energy (Fig. 4(A)) and effective spot size (Fig. 4(B)) had significant effects on ablation cavity depth. Post-hoc Tukey HSD tests confirmed significant pairwise differences among the four tested pulse energies at an effective spot size of 12.8 μ m. For the spot-size comparison, increasing the effective spot size from 35.2 μ m to 64.6 μ m resulted in a statistically significant decrease in cavity depth, while the difference between 12.8 μ m and 35.2 μ m was not statistically significant (p = 0.087).

**Fig. 5.**
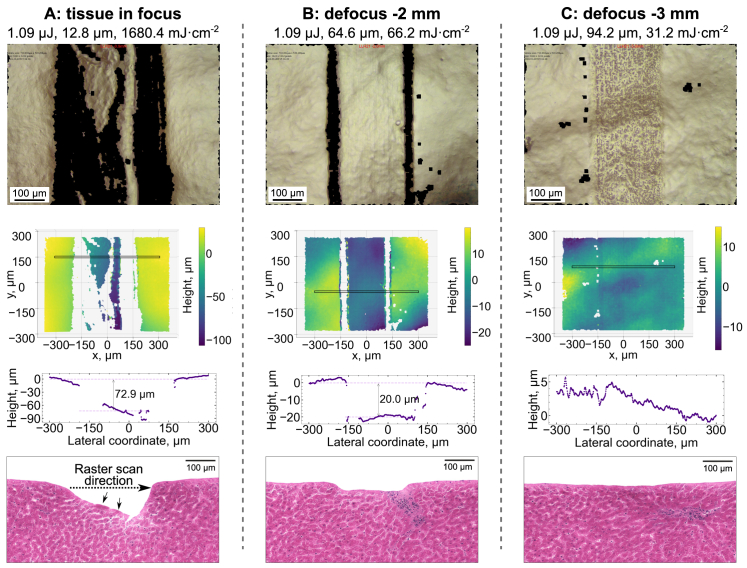
**Examples of cavities ablated in lamb liver using deep-UV femtosecond laser.** Analysis methods, from top to bottom: optical microscopy (top view), surface profilometry (top view heatmap and cross-section of the area in the heatmap marked with black rectangle), histology (cross-section). Black pixels in optical microscopy images, corresponding to transparent pixels in surface profiles, indicate data points missing due to steep gradients or high specular reflection and do not represent the true colour of the tissue. **A**: Irradiation using high energy and small spot size ( D = 12.8 μ m, E = 1.09 μ J, $F_{p}$ = 1680 $\mathrm{mJ}\cdot {\mathrm{cm}}^{-2}$ ) produced a deep cavity with a distorted surface and slope along the scan direction (shown in histology and constant across all examples). Arrowheads mark modified regions in the histology. **B**: Increasing the effective spot size by defocusing the laser beam, while keeping the pulse energy constant, lowers peak fluence ( D = 64.6 μ m, E = 1.09 μ J, $F_{p}$ = 66 $\mathrm{mJ}\cdot {\mathrm{cm}}^{-2}$ ), producing a shallow, uniform ablation cavity, with the cavity bottom closely matching the untreated tissue surface in the optical image. No histological damage is visible. **C**: Below the ablation threshold ( D = 94.2 μ m, E = 1.09 μ J, $F_{p}$ = 32 $\mathrm{mJ}\cdot {\mathrm{cm}}^{-2}$ ), surface profile and histology show no variation after laser irradiation; laser-induced effects are only visible in the optical microscopy (rectangular area with dark brown spots), and are limited to the surface.

Surface profilometry, optical microscopy and histology revealed qualitative differences between the outcomes of ablation performed using different laser parameters. Figure 5 compares cavities created using laser pulses of the same energy (1.09 μ J) but different effective spot sizes. For the smallest spot size (12.8 μ m), achieved by precisely positioning the focus of the laser beam onto the tissue surface, the ablation cavity surface was uneven: it exhibited a slope along the direction of the raster scan (Fig. 5(A)). Under optical microscopy, the cavity surface also appeared rougher compared to the untreated tissue. Moreover, a 15 micron-thick layer of modified tissue (darker colour and denser structure compared to the tissue outside the ablation zone) was visible at the bottom of this cavity in the histology cross-section, which could identify damage caused by applying high peak fluence (1680 $\mathrm{mJ}\cdot {\mathrm{cm}}^{-2}$ ).

Raising the waist of the Gaussian beam above the tissue surface increased the effective spot size on the tissue surface and reduced the peak fluence. Larger effective spot size (64.6 μ m, Fig. 5(B)) produced more uniform cavities with no sign of damage to the tissue surrounding the ablation zone in the histology cross-section. Under these conditions, cavity borders were steeper, no slope was visible, and optical image of the cavity closely matched the untreated tissue surface – in contrast to the cavities obtained using the smallest spot size and maximum fluence (Fig. 5(A)). With the further spot size increase (94.2 μ m), and peak fluence of 32 $\mathrm{mJ}\cdot {\mathrm{cm}}^{-2}$ , which is below the ablation threshold of 39 $\mathrm{mJ}\cdot {\mathrm{cm}}^{-2}$ determined in Section 3.1, laser irradiation did not cause ablation but only produced minor surface modifications visible under optical microscopy (Fig. 5(C)).

**Fig. 6.**
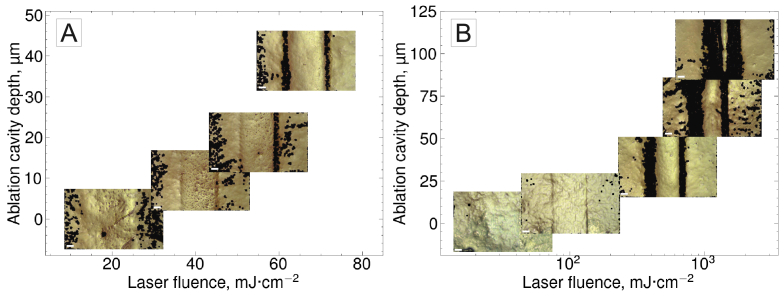
**Optical microscopy images of ablation cavities produced using different laser parameters, shown as data points in the plots of ablation cavity depth vs. peak laser fluence.** Black pixels in the optical microscopy images indicate data points missing due to steep gradients or high specular reflection and do not represent the true colour of the tissue. Scale (white line, bottom left corner of micrographs): 50 μ m. **A**: Effective laser spot size 64.6 μ m. At $F_{p}$ = 66 $\mathrm{mJ}\cdot {\mathrm{cm}}^{-2}$ , the cavity surface is smooth and similar to non-irradiated tissue. With the reduction in irradiation fluence below 60 $\mathrm{mJ}\cdot {\mathrm{cm}}^{-2}$ , ablation cavity depth also decreases, while the density of dark spots on the cavity surface increases. **B**: Effective laser spot size 12.8 μ m. Cavity depth decreases with fluence. Profiles vary from distorted rectangles ( $F_{p}$ >600 $\mathrm{mJ}\cdot {\mathrm{cm}}^{-2}$ ) to uniform rectangles (140–600 $\mathrm{mJ}\cdot {\mathrm{cm}}^{-2}$ ) to shallow lines (40–60 $\mathrm{mJ}\cdot {\mathrm{cm}}^{-2}$ ). Below 40 $\mathrm{mJ}\cdot {\mathrm{cm}}^{-2}$ , the lines have no depth variation and are only visible in the optical microscopy. In contrast to cavities produced by a large laser spot (**A**), no dark spots appear at the cavity bottom.

Surface modifications like those shown in Fig. 5(C) were also observed after tissue irradiation with a defocused laser beam with 64.4 μ m effective spot size, as illustrated in Fig. 6(A). With gradual reduction in laser pulse energy – and therefore in peak fluence – the ablation depth decreased, and the density of dark spots on the ablated cavity surface increased. Surface topography was not altered after irradiation at peak fluence below 40 $\mathrm{mJ}\cdot {\mathrm{cm}}^{-2}$ , however optical surface modifications in the form of dark spots were still present. When the laser beam waist was located on the tissue surface with an effective spot size of 12.8 μ m (Fig. 6(B)), ablation cavity profiles were smooth and free of histological alterations in the peak fluence range of 140 – 600 $\mathrm{mJ}\cdot {\mathrm{cm}}^{-2}$ , corresponding to laser pulse energies between 0.1 μ J and 0.4 μ J. Ablation cavities produced using minimal effective spot size at peak fluence above 600 $\mathrm{mJ}\cdot {\mathrm{cm}}^{-2}$ featured a characteristic slope (similar to Fig. 5(A)). At peak fluence 60 $\mathrm{mJ}\cdot {\mathrm{cm}}^{-2}$ and below, ablation profile appeared as a series of individual lines rather than a single rectangle. Below 40 $\mathrm{mJ}\cdot {\mathrm{cm}}^{-2}$ , lines were only visible in the optical image, and not in the topographical measurements. The results shown in Figs. 5 and 6 confirm that the ablation threshold of lamb liver tissue is around 40 $\mathrm{mJ}\cdot {\mathrm{cm}}^{-2}$ , in agreement with measurements in Section 3.1. Neither dark spots in Fig. 6(A) nor lines in Fig. 6(B) were visible in the histology, so these modifications were surface-limited.

### 3.3. Role of laser pulse repetition rate

**Fig. 7.**
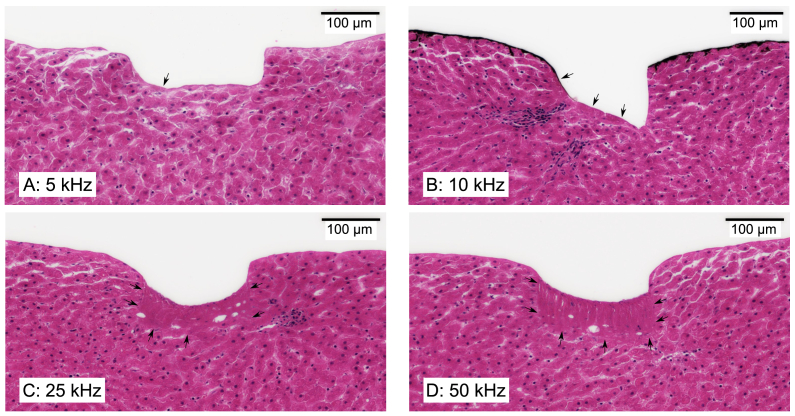
**Histological cross-sections of ablation cavities produced using different laser pulse repetition rates** (laser pulse energy 1.09 μ J, effective spot size 12.8 μ m, peak fluence 1680 $\mathrm{mJ}\cdot {\mathrm{cm}}^{-2}$ , laser spot spatial separation 0.18 μ m): (**A**) 5 kHz, (**B**) 10 kHz, (**C**) 25 kHz, (**D**) 50 kHz. Arrowheads mark isolated areas of modified tissue regions (**A, B**) and borders of the regions with disrupted cellular structure (**C, D**). Black marks on the tissue surface in (**B**) are ink spots that were applied prior to ablation.

To investigate how laser PRR influences tissue ablation, a series of experiments with PRR as the only variable was performed. The laser pulse energy was fixed at 1.09 μ J, with a 12.8 μ m effective spot size (beam waist located on the tissue surface). For each PRR setting (1, 5, 10, 25, 50 kHz), the beam scanning speed was adjusted to maintain the distance between successive laser pulse impact points constant at 0.18 μ m (0.18, 0.9, 1.8, 4.5, 9 mm/s, respectively). Ablation outcomes were assessed using surface profilometry and histological analysis; representative histology results are presented in Fig. 7.

While the cavity surface profiles appeared consistent across the PRRs tested, the corresponding histology results varied significantly. Within the 1 – 5 kHz range, ablation outcomes were similar, with the modified tissue layer at the bottom of the ablation cavity limited to 15 μ m (Fig. 7(A)). At 10 kHz, this layer increased in thickness to 25 μ m (Fig. 7(B)). Further increases in PRR (25 kHz and 50 kHz, Figs. 7(C) and 7(D)) resulted in more pronounced morphological changes: in both cases, a 65-micron layer below the ablated surface featured lack of cellular structure and contained entrapped air bubbles. Notably, these modified regions remained spatially confined to laser-irradiated area, with no observable damage to tissue adjacent on either side. It should be stressed that histological sections may exhibit processing-induced distortion (see Section 2.3), so the thickness values reported here are intended for relative comparison between different samples rather than precise quantitative measurement.

### 3.4. Role of tissue physical properties

The experiments presented above, including analysis of variance across multiple samples, were performed in lamb liver tissue with consistent physical characteristics, including the tissue being brown in colour, moderately rigid and having relatively low moisture content, as qualitatively assessed by visual inspection and palpation. To examine if the ablation outcomes depend on these qualitative tissue characteristics, these experiments were repeated in a different type of lamb liver obtained from the same commercial source and batch: a softer, moister tissue with more noticeable red hue. Throughout this section, these two tissue types are referred to as “Type 1” (rigid, brown tissue used in most experiments) and “Type 2” (soft, red tissue); composition, mechanical and optical properties of these samples were not quantified prior to ablation experiments. A photograph showing the two tissue types side-by-side can be found in Figure S1 of the Supplemental Document.

**Fig. 8.**
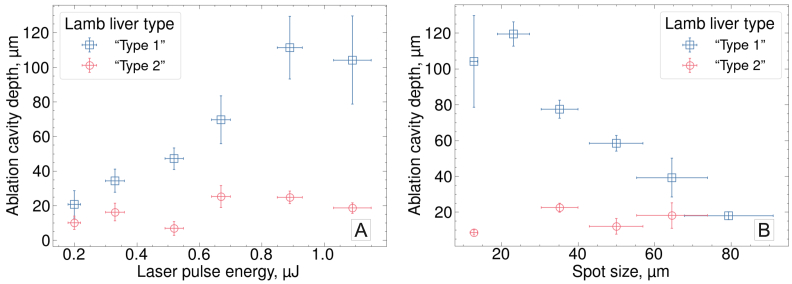
**Comparison of responses of “Type 1” (blue squares) and “Type 2” (red circles) lamb liver tissue to deep-UV ultrashort pulse irradiation. A**: Ablation cavity depth as a function of laser pulse energy (effective spot size fixed at 12.8 μ m). In “Type 1” tissue, cavity depth increases with energy, while in “Type 2” liver it remains nearly constant at ∼ 20 μ m across all the tested energy range. **B**: Ablation cavity depth as a function of effective spot size (laser pulse energy fixed at 1.09 μ J). In “Type 1” tissue, ablation using larger spot sizes results in shallower cavities, whereas in “Type 2” tissue, cavity depth remains around 20 μ m regardless of spot size. Depth measurements were performed in fresh tissue samples immediately after ablation. Each data point represents a single ablation cavity, with vertical error bars showing the standard deviation of the depth measurements within that cavity.

A simplified version of the parameter study from Section 3.2 was conducted, varying either laser pulse energy at a fixed spot size of 12.8 μ m (Fig. 8(A)) or effective spot size at a fixed pulse energy of 1.09 μ J (Fig. 8(B)). The proportional relationship between cavity depth and laser pulse energy observed in “Type 1” liver tissue was not reproduced in the “Type 2” samples (Fig. 8(A)); instead, the ablation depth remained nearly constant at around 20 μ m across all energies. Similarly, unlike in “Type 1” liver tissue, where increasing spot size reduced cavity depth, no such trend was observed in “Type 2” liver (Fig. 8(B)).

**Fig. 9.**
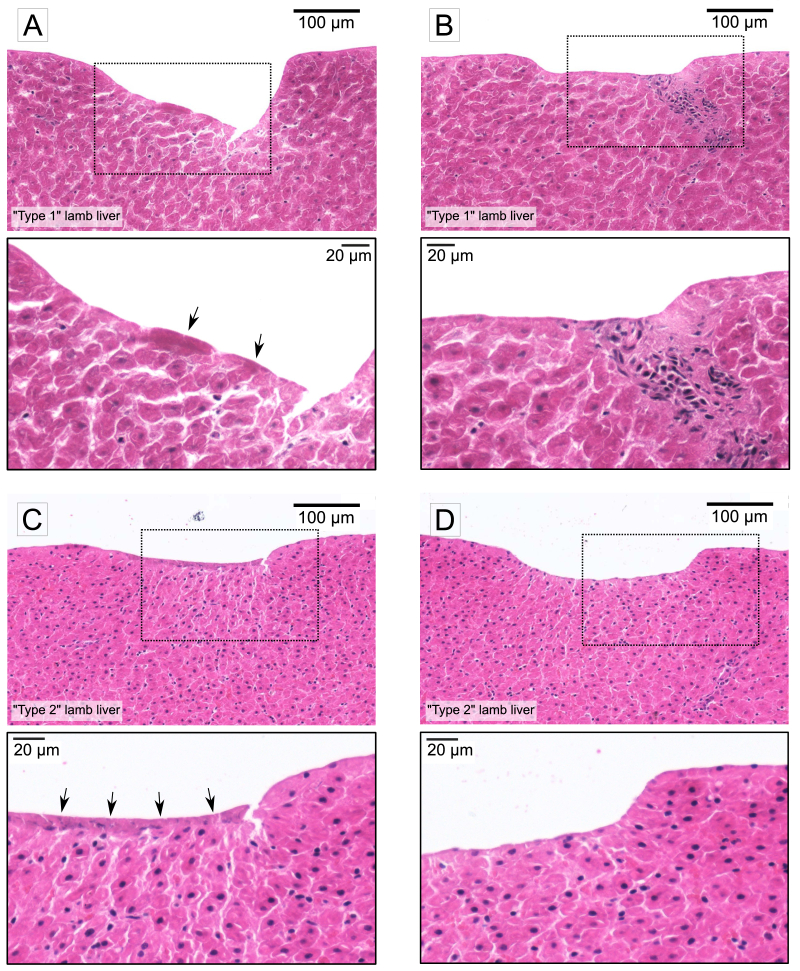
**Histology images of ablation cavities in “Type 1” lamb liver (A, B) and “Type 2” lamb liver (C, D).** For each case, an image with higher magnification is provided below. **A, C**: Laser pulse energy 1.09 μ J, effective spot size 12.8 μ m, peak fluence 1680 $\mathrm{mJ}\cdot {\mathrm{cm}}^{-2}$ . The response of different tissue types to the same laser parameters is different. In “Type 1” tissue, ablation using high fluence results in deep, non-uniform cavity, while in “Type 2” tissue, the cavity depth is much shallower. Tissue modifications, appearing as darker staining on the bottom of the cavity, are present in both tissue types (marked by arrowheads). Within the modified region, distortion of nuclei is observed in “Type 2” tissue (**C**). **B, D**: Laser pulse energy 1.09 μ J, effective spot size 64.6 μ m, peak fluence 66 $\mathrm{mJ}\cdot {\mathrm{cm}}^{-2}$ . Ablation using large spot size and low fluence yields shallow, well-defined cavities with no signs of damage in both tissue types.

As in Section 3.2, histological evaluation offered complementary qualitative insight into tissue morphology after ablation. Figure 9 compares the histology of the cavities ablated in “Type 1” and “Type 2” lamb liver tissue using high peak fluence (1680 $\mathrm{mJ}\cdot {\mathrm{cm}}^{-2}$ , 1.09 μ J pulse energy, 12.8 μ m effective spot size) and low peak fluence (66 $\mathrm{mJ}\cdot {\mathrm{cm}}^{-2}$ , 1.09 μ J pulse energy, 64.6 μ m effective spot size). Although histological dimensions may be affected by processing-induced distortion (see Section 2.3), the sections qualitatively show that low-fluence irradiation at 66 $\mathrm{mJ}\cdot {\mathrm{cm}}^{-2}$ in both tissue types produced well-defined cavities with similar overall morphology (Figs. 9(B), 9(D)). In contrast, high-fluence irradiation (1680 $\mathrm{mJ}\cdot {\mathrm{cm}}^{-2}$ ) yielded drastically different results. In the “Type 1” tissue, high fluence produced an uneven, substantially deeper cavity profile (Fig. 9(A)), while in the “Type 2” tissue the cavity appeared much shallower (Fig. 9(C)). For both lamb liver tissue types, a 15 μ m-thick dark rim of modified tissue was observed beneath the surface ablated with 1680 $\mathrm{mJ}\cdot {\mathrm{cm}}^{-2}$ fluence; in “Type 2” tissue, nuclei within this rim appeared distorted.

To better characterise the qualitative differences between the “Type 1” and “Type 2” lamb liver samples, representative sections from each tissue type were examined using Masson’s trichrome staining (full analysis provided in the Supplemental Document). The “Type 1” tissue contained a slightly higher fraction of collagen-rich areas (22% vs. 17%), a difference that was small but statistically significant (p = 0.0008). This modest increase in relative collagen content is consistent with the firmer tactile impression and may partly contribute to the distinct ablation behaviours observed. The “Type 2” sample also contained more red blood cells, in agreement with its more pronounced red hue prior to fixation. Although mechanical properties were not quantified in this study, previous work has reported correlations between collagen content assessed using trichrome staining and the Young’s modulus of *ex vivo* liver tissue, with firmer tissue exhibiting higher fraction of collagen-rich connection tissue in histology sections [27].

The results of the other ablation experiments (ablation threshold fluence measurements and laser pulse repetition rate studies) were consistent in both types of lamb liver tissue. The ablation threshold fluence was measured to be 37.0 ± 3.0 $\mathrm{mJ}\cdot {\mathrm{cm}}^{-2}$ in “Type 2” tissue, closely matching the value obtained for “Type 1” tissue (Section 3.1). Increasing PRR from 1 kHz to 10 kHz and higher induced subsurface modifications in “Type 2” lamb liver similar to those observed in “Type 1” tissue.

## 4. Discussion

In this work, irradiation of lamb liver tissue with deep-UV femtosecond laser pulses is systematically studied across a range of laser pulse energies, spot sizes, and pulse repetition rates. The regimes of laser-tissue interaction observed in the experiments could be grouped into ablative (tissue removal), non-ablative (tissue surface modification, or deep subsurface disruption of cellular structure), and transition regimes, which combine characteristics of ablative and non-ablative modes. Select sets of experiments were also conducted in lamb liver samples with different physical properties, revealing that the relationship between laser parameters and ablation outcomes can vary significantly even within a single tissue type from the same species.

Identification of laser–tissue interaction regimes that yield optimal ablation outcomes is crucial for the development of clinically useful parameter spaces. In this study, ablation cavities were characterised as “optimal” if they had steep borders, flat surface at the bottom of the cavity and no evidence of adjacent tissue modifications in a histological cross-section (see example in Fig. 5(B)). Maximum depth of the optimal ablation cavities was around 40 microns – it was found that deeper ablation was accompanied by cavity distortion and histological changes (Fig. 5(A)). It is currently unclear whether this specific boundary in the depth value between optimal and non-optimal ablation was influenced by the configuration of the laser scan (for example, by small cavity width or high spatial overlap of laser spots).

**Fig. 10.**
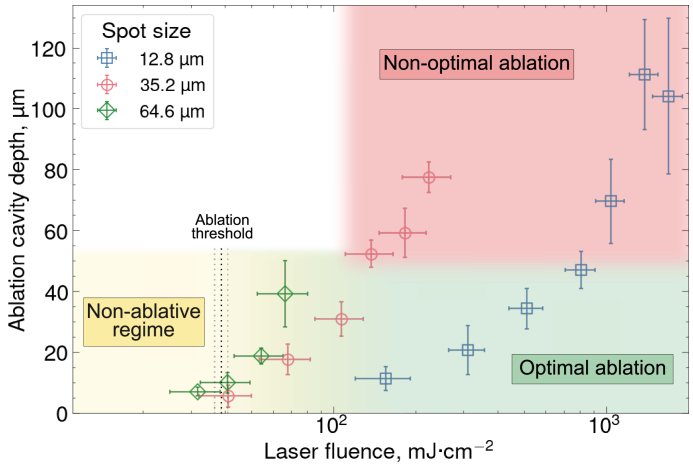
**Ablation cavity depth as a function of peak laser fluence for three effective spot sizes, with identified zones of different laser-tissue interaction regimes.** Data are shown for spot sizes of 12.8 μ m (blue squares), 35.2 μ m (red circles), and 64.6 μ m (green diamonds). For all spot sizes, ablation depth increases with fluence. At the same fluence, deeper cavities were ablated at higher pulse energies (i.e., larger spot sizes). The ablation threshold (38.7 ± 2.1 $\mathrm{mJ}\cdot {\mathrm{cm}}^{-2}$ ) was consistent across all spot sizes. Below this threshold, only non-ablative effects were observed. The fluence range between ~40 $\mathrm{mJ}\cdot {\mathrm{cm}}^{-2}$ and ~60 $\mathrm{mJ}\cdot {\mathrm{cm}}^{-2}$ marked the transition from non-ablative regime to optimal ablation. The upper boundary of optimal ablation regime was ~120 $\mathrm{mJ}\cdot {\mathrm{cm}}^{-2}$ for the 35.2 μ m spot size and ~600 $\mathrm{mJ}\cdot {\mathrm{cm}}^{-2}$ for the 12.8 μ m spot size. For the 64.6 μ m spot size, the non-optimal regime was not reached within the tested fluence range. Each data point represents a single ablation cavity, with vertical error bars showing the standard deviation of the depth measurements within that cavity.

Figure 10 illustrates how ablation cavity depth depends on peak fluence for three spot sizes, highlighting three laser processing windows: optimal ablation, non-optimal ablation, and non-ablative interaction. The optimal ablation window was defined in terms of fluence rather than energy, as the ablation threshold fluence (38.7 ± 2.1 $\mathrm{mJ}\cdot {\mathrm{cm}}^{-2}$ ) was consistent across all effective spot sizes. The upper boundary of this window was approximately 120 $\mathrm{mJ}\cdot {\mathrm{cm}}^{-2}$ for 35.2 μ m spot size and 600 $\mathrm{mJ}\cdot {\mathrm{cm}}^{-2}$ for 12.8 μ m spot size. Beyond this range, histological changes and distortion of cavity profiles were observed (Fig. 5(A)), characteristic of the non-optimal ablation regime. These effects may be attributed to heat accumulation arising from energy deposited to the target tissue in excess of the ablation threshold. Similar histological tissue alterations have been described as thermal damage in porcine liver [28] and porcine intestine [11]. Non-optimal, deep ablation was not observed for the 64.6 μ m spot, likely due to the laser pulse energy limit of 1.09 μ J, resulting in a maximum fluence of only 66 $\mathrm{mJ}\cdot {\mathrm{cm}}^{-2}$ . The lower boundary of the optimal ablation window was around 60 $\mathrm{mJ}\cdot {\mathrm{cm}}^{-2}$ across all spot sizes, and fluence range between 39 $\mathrm{mJ}\cdot {\mathrm{cm}}^{-2}$ and 60 $\mathrm{mJ}\cdot {\mathrm{cm}}^{-2}$ marked a transition between optimal ablation and non-ablative regime.

In the transition region, tissue removal was either inefficient (12.8 μ m effective spot size, Fig. 6(B)) or accompanied by surface-level non-ablative effects (64.6 μ m effective spot size, Fig. 6(A)). This behaviour may be attributed to the Gaussian profile of the laser beam: at fluences close to the ablation threshold, the portion of the beam above the threshold becomes small compared to the size of the Gaussian beam wings below the threshold, or even smaller than the spacing between the raster scan lines (8 μ m). For an effective spot size of 12.8 μ m, this results in ablation of individual lines instead of a rectangular cavity. In the case of larger spot sizes (35.2 μ m and 64.6 μ m) with high spatial overlap, the lower-intensity wings of the beam contribute to the laser-tissue interaction disproportionally, which may lead to cumulative thermal effects. However, given the shallow penetration depth of 206 nm radiation into the tissue and thermal confinement of ultrashort laser pulses, these effects were superficial.

The threshold peak fluence observed in this work (38.7 ± 2.1 $\mathrm{mJ}\cdot {\mathrm{cm}}^{-2}$ ) is consistent with values reported for deep-UV ultrashort laser ablation of other soft tissues, including porcine cornea (60 $\mathrm{mJ}\cdot {\mathrm{cm}}^{-2}$ at 206 nm, 290 fs [17]), and bovine brain (22 $\mathrm{mJ}\cdot {\mathrm{cm}}^{-2}$ ± 50% at 211 nm, 30 ps [20]). Notably, the threshold reported here is around ten times lower than that determined for a similar tissue type (porcine liver) using NIR femtosecond pulses (300 $\mathrm{mJ}\cdot {\mathrm{cm}}^{-2}$ at 775 nm, 150 fs [28]). Definitions of fluence (e.g., whether peak or average fluence is reported) may vary across studies, which should be considered when comparing values. Still, an order of magnitude difference between characteristic ablation thresholds for NIR and deep-UV femtosecond lasers highlights the ability of the latter to precisely remove tissue with significantly less laser energy.

The minimal cavity depth that could be confidently measured in the examined soft tissue model was 10 microns, achieved using a 12.8 μ m effective spot size and 0.1 μ J laser pulse energy (corresponding to 160 $\mathrm{mJ}\cdot {\mathrm{cm}}^{-2}$ peak fluence). While this value exceeds the sub-micrometre depth of ablation cavities produced using 193 nm nanosecond [29,30] and 206 nm femtosecond lasers [17] in corneal tissue, it is still well below the axial precision of conventional surgical tools like electrocautery, ultrasonic aspirator, and long-pulsed lasers [11,31,32]. In the examined soft tissue samples, natural variations in the surface topography routinely exceeded 10 μ m, which limited the ability to resolve shallower cavities. Additionally, the laser was scanned with very high spatial overlap of laser spots (0.18 μ m spacing, corresponding to ~99% overlap for the 12.8 μ m spot size). Under these conditions, the 10 μ m cumulative depth may reflect sub-micron contributions from individual pulses. While greater axial ablation precision might be achievable by increasing the laser spot spatial separation, validating this would require higher-resolution surface profiling.

Temporal pulse overlap, determined by the laser pulse repetition rate, was found to strongly influence ablation outcomes. As discussed in Section 3.3, irradiation of soft tissue with laser pulses at repetition rates above 10 kHz resulted not only in tissue removal, but also in disruption of cellular structure up to 65 μ m below the cavity surface – well beyond the typical penetration depth of 206 nm radiation [6]. Importantly, no discolouration or tissue morphological changes were observed around the irradiation zone: the modified regions were confined to areas directly below the laser-irradiated surface. Although these observations are not sufficient to determine the mechanisms underlying tissue modifications induced by deep-UV laser pulses at high PRRs, they are consistent with a hypothesis that pulse-to-pulse mechanical interactions may contribute to subsurface disruption.

Since each ablation event generates a recoil pressure wave [6], pulses arriving within the lifetime of these waves may interact with residual stress fields in the tissue, particularly when successive laser pulse impact points are not resolved spatially. In the reported experiments, performed at high spatial overlap between laser spots, the onset of subsurface disruption at 10 kHz could reflect the timescale of mechanical relaxation following a singular ablation event. The absence of signs of thermal damage around the irradiated zone in the histology suggests that heat accumulation is unlikely to be the main mechanism behind the observed tissue modifications, as this pattern is inconsistent with typical diffusion-driven heat deposition reported during thermal laser ablation using longer pulse durations and wavelengths [8,33,34]. However, dedicated real-time diagnostics (acoustic and/or thermal) would be required to determine the tissue damage mechanism at high PRRs definitively, and alternative explanations cannot be ruled out at this stage. It is important to note that these high-PRR experiments were conducted using laser parameters that produced non-optimal ablation at 1 kHz. Further investigation is needed to determine whether similar effects occur in optimised ablation regimes.

Although disruption of cellular structure by irradiation at high laser PRR was an undesired side effect in the context of tissue removal, it may nonetheless be of clinical relevance. The cavitron ultrasonic surgical aspirator (CUSA), a commonly used tool for tumour resection, relies on high-frequency mechanical vibrations (23 kHz or 36 kHz) to fragment or emulsify malignant tissue. The typical precision of CUSA is limited to millimetre scale, with a ~100 μ m shrunken zone surrounding the ablation cavity [31]. In contrast, cellular disruption induced by deep-UV ultrashort laser pulses at PRRs above 10 kHz, as demonstrated in Section 3.3, achieved sub-millimetre lateral precision with no detectable damage beyond the irradiation zone, surpassing the precision of CUSA. This laser-tissue interaction regime presents a promising candidate for further investigation. However, comparable effects achieved using similar laser parameters in the more conventional NIR wavelength range have been reported [11]; the differences between NIR and deep-UV radiation in this particular laser-tissue interaction regime remain to be explored.

An important feature of this study was the use of a soft tissue model with low collagen content. To the authors’ knowledge, with the exception of the work by Bille *et al.* [20], most studies on deep-UV laser ablation of soft tissue using nanosecond to femtosecond pulses were performed in corneal tissue [17,18,35–38], which is rigid and mechanically robust [6], enabling higher ablation precision. As shown in Section 3.4, physical properties of the target material can significantly influence the ablation outcome. In one of the two tested tissue types, no proportional relationship between ablation depth and laser pulse energy – described in Section 3.2 of the current work and previously reported for porcine cornea [17,38] and brain [20] – was observed. It was also found that irradiation regimes characterised as optimal in the main parameter study provided precise, damage free ablation in both lamb liver tissue types, in contrast with the non-optimal, high-fluence irradiation. Although a formal assessment of mechanical, optical, or chemical properties was beyond the scope of this study, histological analysis of the two lamb liver tissue types indicated their clear compositional differences (notably in the relative fraction of collagen-rich connective tissue and red blood cells count), which were consistent with the qualitatively assessed physical properties of the two lamb liver tissue types and may explain their differing ablation behaviour. These comparative findings are particularly relevant in the context of clinical translation of the proposed tissue removal method. The differences in tissue response to deep-UV femtosecond laser irradiation underscore the need for further investigation of ablation across tissues with diverse mechanical, viscoelastic, and optical properties, characterisation of these properties, and relating them to the ablation outcomes, with the emphasis on more delicate and clinically relevant tissues such as brain.

## 5. Application prospects and conclusions

This study systematically explored ablation of soft biological tissue (lamb liver) using deep-UV femtosecond laser pulses – a theoretically promising approach for precise tissue removal that combines the micrometre-scale penetration of deep-UV light into biological tissues with the reduced collateral damage of ultrashort pulses. Despite this potential, applications of deep-UV femtosecond lasers beyond corneal reshaping remain largely unexplored.

By systematically varying laser pulse energy and spot size, processing windows that enabled precise, damage-free tissue removal, were identified. Within these windows, ablation with ~10 μ m precision was achieved, surpassing typical values for conventional surgical tools and highlighting the clinical relevance of this regime. The ablation threshold of lamb liver tissue measured in this study (38.7 ± 2.1 $\mathrm{mJ}\cdot {\mathrm{cm}}^{-2}$ ) is consistent with previously reported values for similar irradiation parameters. Clear differences were observed in the response of tissues with different physical properties to the same laser parameters, emphasising the need to study a broader range of soft biological tissues and to formally characterise their physical properties.

These findings expand the currently limited understanding of deep-UV ultrashort pulse ablation in soft biological tissues and demonstrate its potential beyond applications in ophthalmology. In the broader landscape of laser surgery, deep-UV ultrashort pulses could occupy a distinct regime. Microsurgery at the scale demonstrated in this work is not achievable with photothermal ablation using longer pulse durations, where the absence of thermal and stress confinement leads to heat diffusion, collateral coagulation and carbonisation, and poor axial control [6,8,33,39]. Likewise, ultrashort-pulse ablation in the near-infrared with clinically relevant spot sizes (10–40 μ m) typically produces cavity depths exceeding 20 μ m and thermal damage ranging from a few micrometre [26,40] to tens of micrometres [11,41], even under optimised conditions.

“Cellular-level” tissue resection precision theoretically achievable by deep-UV ultrashort pulses is comparable to that of high-NA, plasma-mediated NIR femtosecond ablation, which can reach sub-micrometre accuracy [16]. However, the ablation mechanisms differ fundamentally. Plasma-mediated NIR ablation relies on nonlinear absorption and can produce subsurface effects in transparent or weakly scattering tissues, making it uniquely suited for cellular and subcellular manipulation [42]. The examples of *in vivo* NIR femtosecond laser microsurgery include resection of neuron cell bodies and fibres in *C. elegans* [16,42], modification of *Drosophila* embryonal tissue [43], and controlled disruption of blood vessels in rodent brain [44]. The same nonlinear absorption that enables this high spatial precision also introduces practical constraints: high-NA focusing restricts the working distance, and axial positioning must be controlled on the sub-micrometre scale, since small focus shifts can suppress ablation or cause it to occur in undesired location [16,42].

Deep-UV ultrashort-pulse ablation, by contrast, is governed by strong linear absorption and is therefore intrinsically surface-confined; light penetration in tissue is further limited by femtosecond-scale pulse duration. Ablation occurs once the fluence exceeds a certain threshold and does not require plasma generation. This mechanism allows the same micrometre-scale axial precision to be maintained across a wide range of spot sizes, from single-cell scales to larger surgically relevant beams, and makes the process insensitive to small variations in tissue surface or minor defocusing. Consequently, the most compelling clinical niche of deep-UV femtosecond laser ablation is as a precise surface-removal tool, particularly as a “clean-up” modality following bulk resection, where it could enable controlled removal of residual micrometre-scale tumour margins while minimising risk to adjacent critical structures. Recent advances in high-brightness, high-beam quality deep-UV ultrashort pulsed laser sources and specialised fibre technology make such clinical applications potentially more achievable [45,46].

It is important to acknowledge the limitations of this work. Laser ablation was performed with an intentionally high laser spot spatial overlap, a setting that may have contributed to the accumulation of thermomechanical effects which led to non-optimal, unsatisfactory ablation outcomes. Nonetheless, even under these constrained conditions, processing windows that yielded precise, damage-free tissue removal could still be defined. In a clinical setting, pulse spacing would likely be increased to enable faster resection; however, the optimal ablation regimes identified in this study, despite being less efficient, could potentially be safer in case of scanning equipment malfunctioning. Another limitation is that ablation was studied in store-bought lamb liver, which is not a clinically relevant tissue model; therefore, these findings cannot yet be directly translated to clinical applications.

Future work will focus on increasing the efficiency of tissue removal while maintaining the high axial and transverse precision demonstrated in this study. This will involve exploring the effects of pulse repetition rate and laser spot spatial separation within the regimes identified as optimal, with the aim of achieving faster scanning speeds and improved axial precision, and extending these parameter scans to high-fluence (non-optimal) regimes to better distinguish single-pulse ablation effects from potential cumulative thermal and mechanical interactions. In parallel, future studies will extend to clinically relevant tissue models. Preliminary proof-of-concept experiments performed in a small set of fresh *ex vivo* porcine brain samples indicate that the deep-UV ultrashort laser pulse parameters optimised in lamb liver model can produce well-defined cavities in brain tissue [47]. Building on these initial findings, future experiments will involve a systematic parameter investigation in brain tissue and evaluation of deep-UV ultrashort-pulsed laser ablation in human glioblastoma samples.

## Supplemental information

Supplement 1 Tissue composition analysis using Masson's trichrome histology stain https://doi.org/10.6084/m9.figshare.30772700

Visualization 1 Footage of the ablation plume produced by deep-UV femtosecond laser in ex vivo lamb liver tissue, captured by event-based (neuromorphic) camera. Ablation is performed at 1 kHz pulse repetition rate, with the tissue fixed under the laser beam (ablatio https://doi.org/10.6084/m9.figshare.30126943

Visualization 2 Footage of the ablation plume produced by deep-UV femtosecond laser in ex vivo lamb liver tissue, captured by event-based (neuromorphic) camera. Ablation is performed at 1 kHz pulse repetition rate, with the tissue fixed under the laser beam (ablatio https://doi.org/10.6084/m9.figshare.30126937

Visualization 3 Footage of the ablation plume produced by deep-UV femtosecond laser in ex vivo lamb liver tissue, captured by event-based (neuromorphic) camera. Ablation is performed at 1 kHz pulse repetition rate, with the tissue fixed under the laser beam (ablatio https://doi.org/10.6084/m9.figshare.30126946

## Data Availability

The data that support the findings of this study are available from the corresponding author upon reasonable request.

## Supplemental document

See Supplement 1 for supporting content.
