# Supplementary material for: Deep ultraviolet ultrashort laser pulses for precise ablation of soft biological tissue
Source: Biomed Opt Express. 2025 Dec 10;17(1):183–201. doi: 10.1364/BOE.578629 (PMC12795447; doi:10.1364/BOE.578629)
Supplement: Supplement 1 [file boe-17-1-183-s001.pdf]

Supplemental Document

Biomedical Optics EXPRESS

Deep ultraviolet ultrashort laser pulses for precise ablation of soft biological tissue: supplement

TATIANA K. MALIKOVA,[1,*] RAINER J. BECK,[1] TIMOTHY P. FRAZER,[1] PAUL M. BRENNAN,[2] KEVIN DHALIWAL,[3] ROBERT R. THOMSON,[1] AND JONATHAN D. SHEPHARD[1]

[1] *Institute of Photonics and Quantum Sciences, Heriot-Watt University, Edinburgh, EH14 4AS, UK*
[2] *Translational Neurosurgery, Centre for Clinical Brain Sciences, University of Edinburgh, Edinburgh, EH16 4SB, UK*
[3] *Centre for Inflammation Research, Institute for Regeneration and Repair, University of Edinburgh, Edinburgh, EH16 4UU, UK*
[*] *tkm2000@hw.ac.uk*

This supplement published with Optica Publishing Group on 10 December 2025 by The Authors under the terms of the Creative Commons Attribution 4.0 License in the format provided by the authors and unedited. Further distribution of this work must maintain attribution to the author(s) and the published article's title, journal citation, and DOI.

Supplement DOI: https://doi.org/10.6084/m9.figshare.30772700

Parent Article DOI: https://doi.org/10.1364/BOE.578629

Deep ultraviolet ultrashort laser pulses for precise ablation of soft biological tissue: supplemental document

1. VISUAL COMPARISON OF LAMB LIVER TISSUE SAMPLES

The experiments described in Section 3.4 of the Manuscript were performed using two types of *ex vivo* lamb liver tissue obtained from the same commercial source and batch. The samples were distinguished based on qualitative visual and tactile assessment and are referred to as “Type 1” (firmer tissue with lower apparent moisture content, brown in colour) and “Type 2” (softer, moister tissue with a more pronounced red hue). These labels are used solely to differentiate the two visually and tactilely distinct sample types; the mechanical, optical or chemical properties were not quantitatively assessed. Figure S1 shows representative photographs of the two tissues side-by-side prior to ablation experiments.

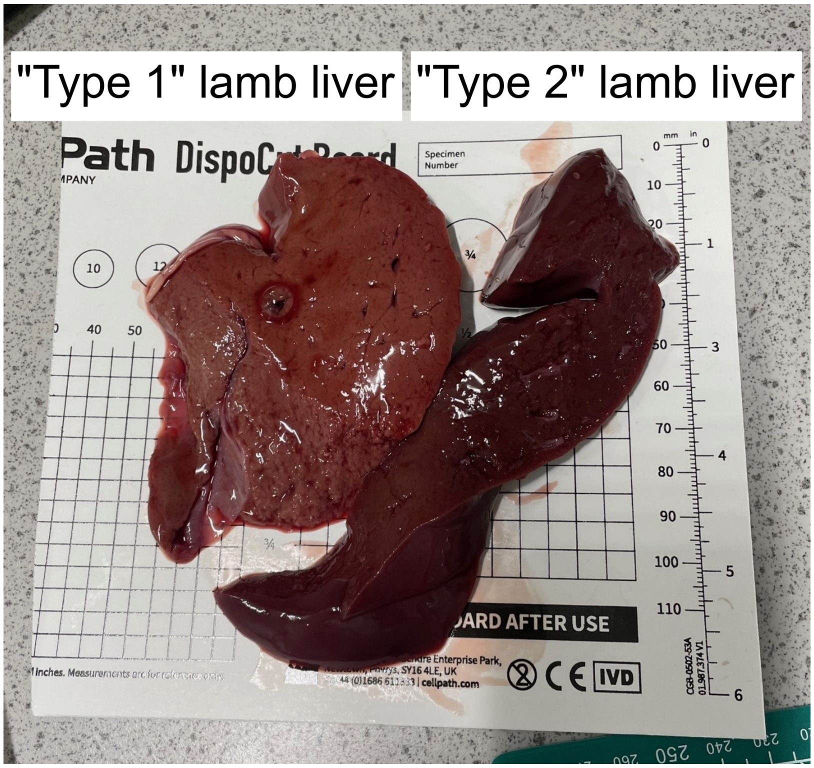

Fig. S1. Representative “Type 1” and “Type 2” lamb liver tissue samples before the experiments.

2. ANALYSIS OF LAMB LIVER TISSUE COMPOSITION USING MASSON’S TRICHROME STAIN

To characterise the qualitative differences between the “Type 1” and “Type 2” lamb liver samples, one section from each tissue type was stained using Masson’s trichrome (the preceding histology protocol is provided in Section 2.3 of the Manuscript). Masson’s trichrome distinguishes cell nuclei (dark purple/black), cytoplasm (magenta), and collagen (blue), and has previously been

used to evaluate relative collagen content in bovine liver tissue in studies correlating histological composition with mechanical properties (Young's modulus and fracture toughness) [1].

The stained sections were scanned using a high-resolution optical microscope and analysed in QuPath software. Representative regions for the "Type 1" and "Type 2" tissues are shown in Figure S2. Qualitative differences were apparent: in the "Type 2" tissue, nuclei were more prominent, and red blood cells were more abundant between liver cells (hepatocytes), consistent with this tissue type's red hue prior to fixation.

Pixel classification in QuPath (artificial neural network approach) was used to estimate the relative fractions of collagen fibres and hepatocytes. Figure S2 includes the resulting segmentation masks (yellow for liver cells, blue for collagen fibres) alongside the original images.

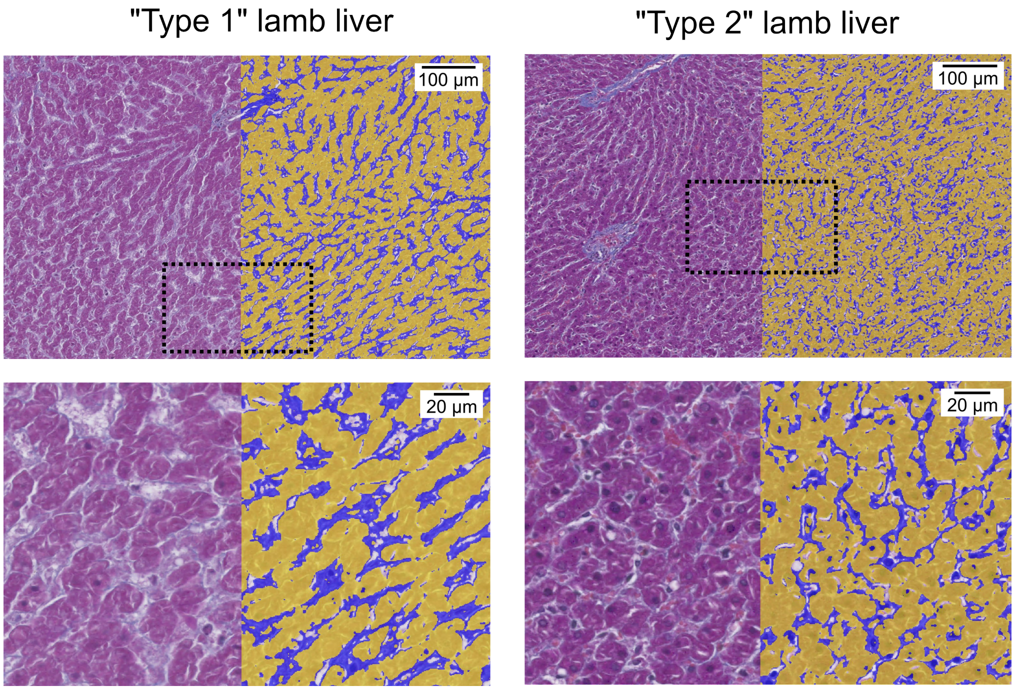

Fig. S2. Masson's trichrome staining of "Type 1" (left) and "Type 2" (right) lamb liver sections at lower (top) and higher (bottom) magnification. Dashed rectangles in the top images mark the regions shown in the close-up images below. In each image, the left panel shows the original microscopy image and the right panel shows the pixel classification mask of the adjacent region (liver hepatocyte cells in yellow, collagen in blue). Compared with the "Type 1" tissue, the "Type 2" sample exhibits more prominent nuclei and higher count of red blood cells between hepatocytes.

To quantitatively assess collagen content in the two histology samples, the following analysis workflow was used:

1. 50 square regions (500 × 500 µm) were selected from each histology section, avoiding large blood vessels (cavities surrounded by dense collagen fibres).

2. The QuPath pixel-classification algorithm was applied to each region to quantify the areas occupied by collagen fibres, liver cells, and background pixels. The collagen fraction was calculated as the ratio of collagen-positive area to total region area.

3. One-way ANOVA (significance level $p = 0.05$; Python `statsmodels`) was performed on the collagen-fraction measurements, and medians with median absolute deviations were calculated for each sample.

The results are presented in Figure S3 as violin plots. The median collagen fraction in the "Type 1" sample was 22.1 ± 2.1% (median absolute deviation), compared to 17.3 ± 2.6% in the "Type 2" sample. ANOVA confirmed that tissue type had a significant effect on collagen fraction (p =

0.000806). These findings are consistent with the earlier qualitative impression that the “Type 1” tissue was firmer and more mechanically strong.

Yarpuzlu *et al.* [1] reported that, in *ex vivo* bovine liver, increases in Young’s modulus and fracture toughness during preservation were correlated with increases in the fraction of tissue stained positive for collagen with Masson’s trichrome. The collagen fractions in commercially sourced lamb liver samples measured in the present study fall within the range reported for bovine liver one day after harvesting. While the histological analysis presented here does not replace direct mechanical or viscoelastic measurements, it provides a more objective assessment of compositional differences between the two tissue types than visual or tactile inspection alone.

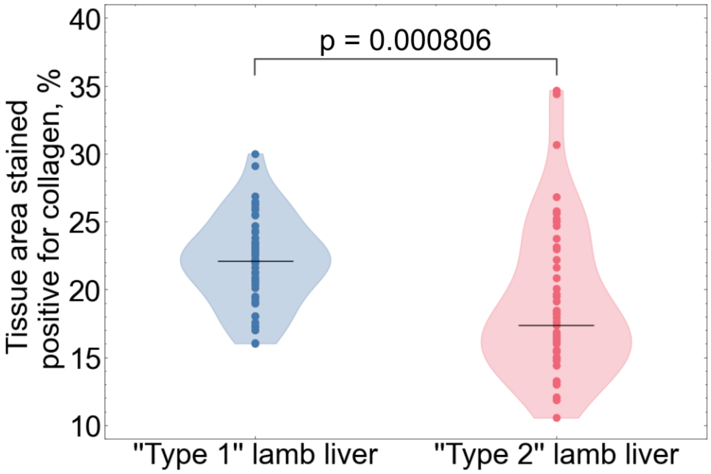

Fig. S3. Collagen fraction in “Type 1” and “Type 2” lamb liver samples. Each data point corresponds to the collagen fraction measured in one 500 × 500 μm region. Black lines indicate sample medians. The median collagen fraction was 22.1 ± 2.1% in the “Type 1” sample and 17.3 ± 2.6% in the “Type 2” sample; the difference is statistically significant ($p = 0.000806$, one-way ANOVA).

REFERENCES

1. B. Yarpuzlu, M. Ayyildiz, O. E. Tok, *et al.*, “Correlation between the mechanical and histological properties of liver tissue,” J. Mech. Behav. Biomed. Mater. **29**, 403–416 (2014).
